# Promising outcomes of the PD-L1 inhibitor Socazolimab in recurrent and metastatic cervical cancer: a case report

**DOI:** 10.3389/fonc.2025.1541760

**Published:** 2025-03-24

**Authors:** Zichao Xu, Minjie Wang, Long Gong, Guanghong Luo, Yucong Zhang, Yayan Zhou, Zihuang Li

**Affiliations:** The Second Clinical Medical College of Jinan University, Department of Radiotherapy, Shenzhen People’s Hospital, Shenzhen, Guangdong, China

**Keywords:** advanced cervical cancer, immune checkpoint inhibitor, PD-L1, Socazolimab, case report

## Abstract

Recurrent and metastatic advanced cervical cancer carries a poor prognosis, and there is currently no standardized treatment regimen. Immunotherapy, as an emerging and effective treatment strategy, has attracted significant attention. Socazolimab, a programmed death ligand 1 monoclonal antibody developed in China, is primarily used to treat patients with recurrent or metastatic cervical cancer who have failed or are intolerant to first-line platinum-based chemotherapy, regardless of programmed death ligand 1 expression status. We report the case of a Chinese patient with advanced cervical cancer and multiple metastases, who showed significant tumor regression after receiving Socazolimab treatment. Currently, during ongoing immunotherapy, the tumor continues to demonstrate persistent remission, with no new lesions observed. This case suggests that Socazolimab may offer a promising therapeutic option for patients with metastatic cervical cancer. In this report, the safety profile of Socazolimab was favorable, with no severe or uncontrollable adverse events. This case provides compelling evidence of the antitumor activity and manageable toxicity of Socazolimab in metastatic cervical cancer patients, offering valuable insights for further research and clinical application of the drug.

## 1 Introduction

Cervical cancer is the fourth most common cancer among women worldwide and is also one of the top three cancers affecting women under the age of 45 (1). Standard treatment options for early-stage and locally invasive cervical cancer include radical hysterectomy, radical trachelectomy, and pelvic lymphadenectomy, combined with concurrent chemoradiotherapy (2). For patients with recurrent and metastatic advanced cervical cancer, chemotherapy-based systemic treatment is typically the cornerstone of therapy (3). However, due to the poor prognosis in advanced-stage patients and the significant adverse effects associated with existing therapies, there is an urgent need to identify new treatment options. In recent years, immune checkpoint inhibitors (ICIs) have rapidly emerged as a leading form of cancer immunotherapy and have demonstrated significant clinical efficacy (4, 5). In particular, ICIs targeting programmed death-1 (PD-1), programmed death-ligand 1(PD-L1), and cytotoxic T-lymphocyte-associated protein 4 (CTLA-4) have become some of the most promising therapeutic approaches in oncology (6–8).

Socazolimab is a monoclonal antibody targeting PD-L1 that has been included in the breakthrough therapy drug review process by the China National Medical Products Administration (NMPA). It is primarily used to treat patients with recurrent or metastatic cervical cancer who have failed or are intolerant to first-line platinum-based chemotherapy, regardless of PD-L1 expression status (9). In 2024, the Chinese Society of Clinical Oncology (CSCO) included Socazolimab (PD-L1) in the ‘Cervical Cancer Diagnosis and Treatment Guidelines 2024,’ recommending it as a ‘systemic therapy for recurrent or metastatic cervical cancer (Level 2B).’ This article presents the case of a patient with recurrent and metastatic cervical cancer who was treated with Socazolimab.

## 2 Case description

In April 2008, a 46-year-old married and parous Chinese woman with no significant medical history presented with unexplained vaginal bleeding. After undergoing relevant examinations at a local hospital, she was diagnosed with cervical cancer. In May, she underwent radical total hysterectomy, bilateral salpingo-oophorectomy, and pelvic lymphadenectomy. Postoperative pathology revealed squamous cell carcinoma of the cervix with minimal local invasion (stage I-II), and no lymph node or distant organ metastases were observed. The patient did not receive postoperative chemoradiotherapy and did not have regular follow-up examinations.

**Figure 1:**
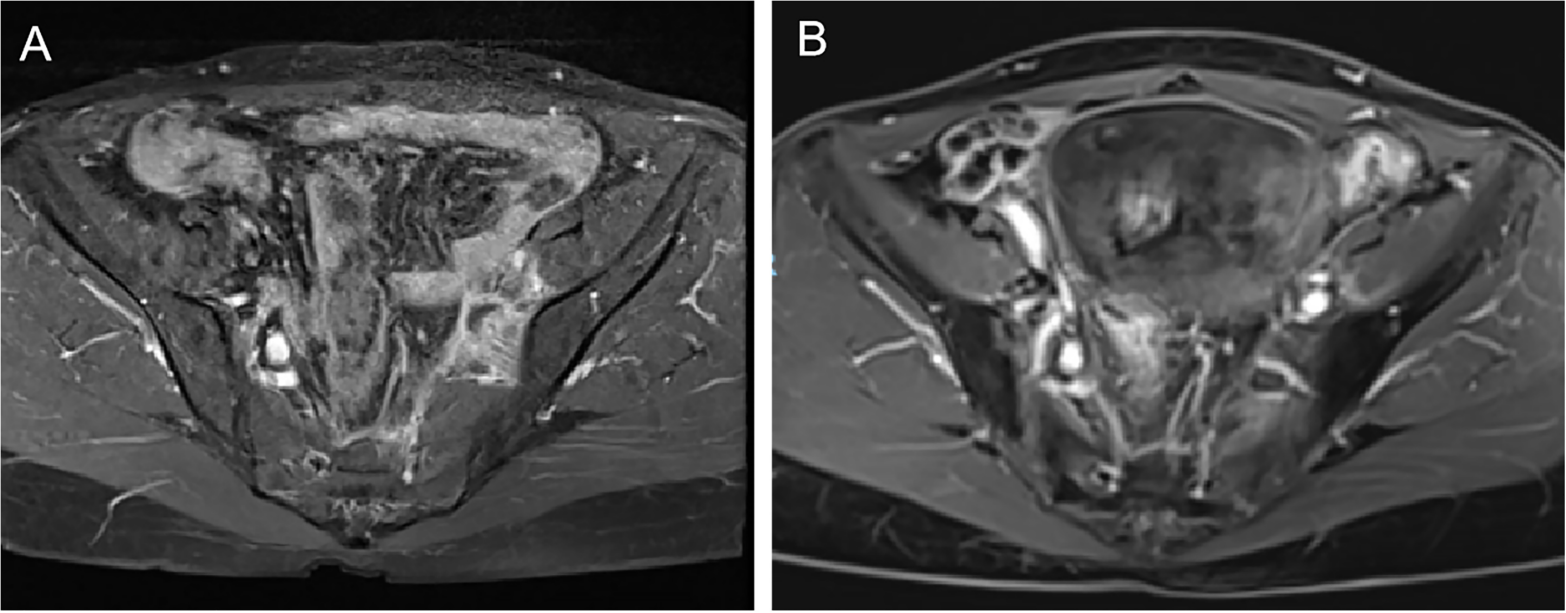
**(A)** pre-radiotherapy imaging results for the left iliac vascular region. **(B)** post-radiotherapy imaging results for the left iliac vascular region.

In August 2019, the patient sought medical attention due to lower limb edema. Chest and abdominal CT scans revealed metastasis to the left iliac vascular region, as well as lymph node involvement in the left iliac vessels and mesorectum. In September 2019, the patient started a treatment regimen consisting of six cycles of paclitaxel plus carboplatin chemotherapy, combined with apatinib. After the fourth cycle, evaluation showed partial remission (PR). However, following the fifth and sixth cycles, a follow-up CT scan revealed no significant reduction in the tumor, and the disease was classified as stable (SD). From March to April 2020, the patient underwent two cycles of albumin-bound paclitaxel, cisplatin, and bevacizumab combination chemotherapy and targeted therapy. A CT scan in April 2020 showed no changes in the tumor.In July 2020, the patient received radiotherapy with 6MV X-ray radiation and Volumetric Modulated Arc Therapy (VMAT). The prescribed doses were as follows: PTV-High 95% volume: 47.5Gy/25 fractions (F); PTV-para 95% volume: 45Gy/25F; PGTV (pelvic wall recurrence site) 95% volume: 61.25Gy/25F. The treatment was completed successfully, and follow-up examinations showed complete remission (CR) of the recurrent lesions (**Figure 1**).

In August 2023, the patient developed an unexplained dry cough and subsequently underwent PET-CT and contrast-enhanced CT at the hospital. Imaging revealed new metastatic lesions in the right upper lung lobe, as well as multiple lymph node metastases in the mediastinum, right lung hilum, and other regions. Severe external compression and critical stenosis of the right main bronchus were also observed. At that time, the patient declined treatment due to personal reasons. However, in May 2024, she returned to the hospital with worsening symptoms. Chest contrast-enhanced CT showed further progression of the metastatic lesion in the right upper lung lobe, along with enlargement of the mediastinal and right hilar lymph nodes, which had merged with the tumor. Additionally, a newly enlarged right supraclavicular lymph node was observed, suspected to be metastatic. The enlarged mediastinal and right hilar lymph nodes were also found to be invading the superior vena cava.

**Figure 2:**
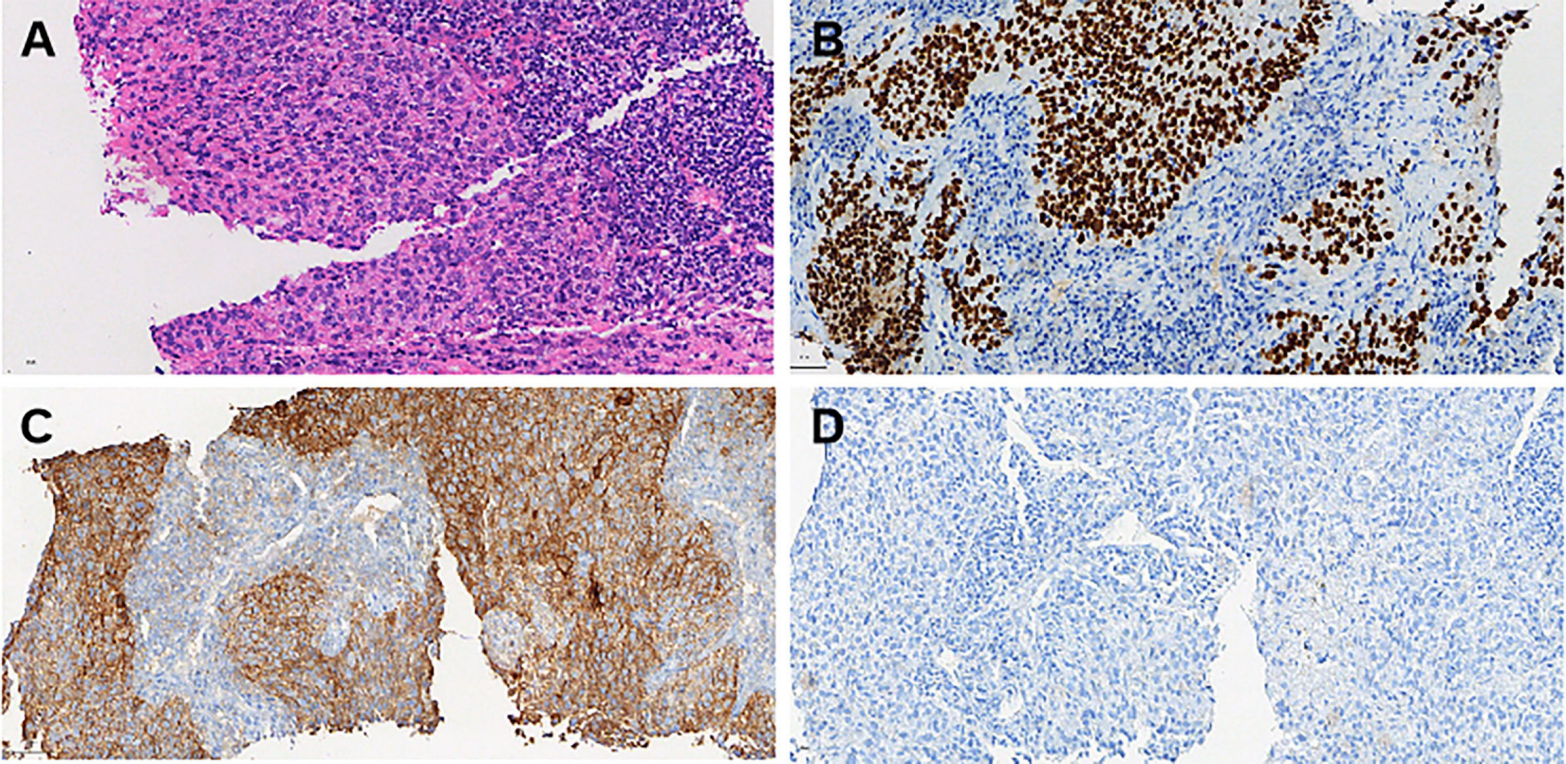
Histopathological and immunohistochemical findings from the cervical lymph node biopsy (May 2024). **(A)** Hematoxylin and eosin (H&E) staining: The metastatic squamous cell carcinoma (SCC) tissue exhibits pleomorphic tumor cells with hyperchromatic nuclei and irregular architecture. The tumor is arranged in solid nests with prominent keratinization. Scale bar = 50 μm (magnification: 40×). **(B)** p40 immunohistochemical staining: Strong and diffuse nuclear positivity for p40 confirms the squamous cell carcinoma phenotype of the metastatic lesion. Scale bar = 50 μm (magnification: 40×). **(C)** PD-L1 (22C3) immunohistochemical staining: Tumor cells demonstrate strong membranous PD-L1 expression with a tumor proportion score (TC) of 80%, indicating potential responsiveness to immune checkpoint blockade therapy. Scale bar = 50 μm (magnification: 40×). **(D)** PD-L1 negative control: Negative control staining performed under the same conditions shows the absence of PD-L1 expression in non-tumoral regions, confirming staining specificity. Scale bar = 50 μm (magnification: 40×).

The patient underwent a cervical lymph node (right supraclavicular lymph node) biopsy in May 2024, and pathology confirmed metastatic squamous cell carcinoma. Immunohistochemical(IHC) analysis revealed the following results: p16 (diffusely strong +), MSH2 (+), MSH6 (+), PMS2 (slightly weak +), MLH1 (+), Her-2 (0), PD-L1 (BP6099, TC score: 80%), PD-L1 Neg (–), p63 (+), CK5/6 (+), p40 (+), CD5 (–), CD117 (–). *In situ* hybridization for EBER was negative (**Figure 2**). The patient was diagnosed with multiple metastases (lung, mediastinum, and cervical lymph nodes) following pelvic recurrence of cervical cancer after chemoradiotherapy.

**Figure 3:**
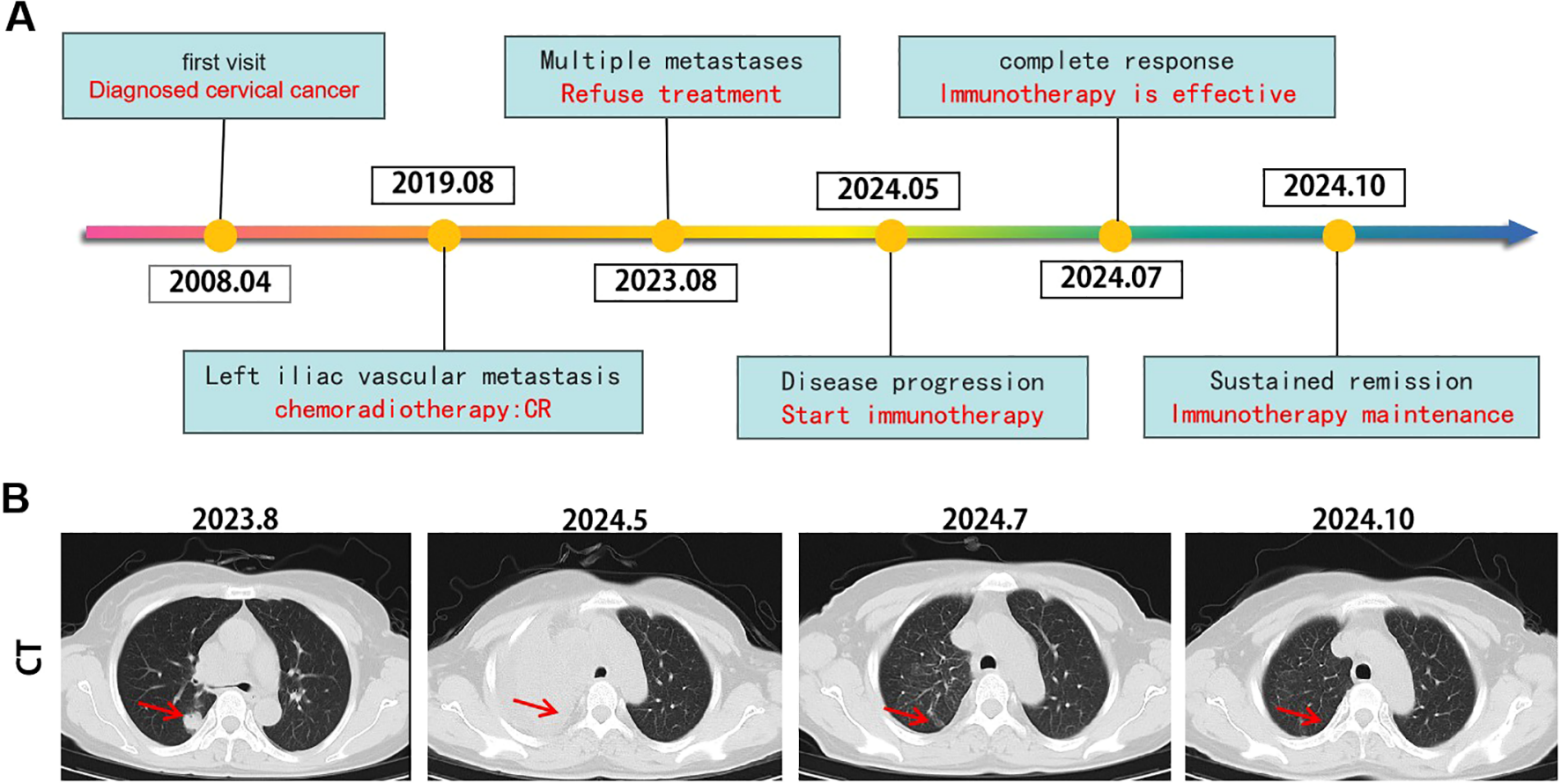
Tumor responses after therapy. **(A)** Timeline of disease status and corresponding treatment regimens. **(B)** Time line of treatments of the patient and changes in CT of lung during treatments.

On May 20, 2024, the patient began receiving Socazolimab 200 mg every three weeks as part of her immunotherapy regimen. The treatment proceeded smoothly, with no significant immune-related adverse events observed. After completing the planned course of immunotherapy, a follow-up CT scan in July 2024 (**Figure 3**) revealed significant improvement in the consolidation of the right upper lung lobe, with the bronchus largely patent. The metastatic lymph nodes in the mediastinum, right supraclavicular fossa, and right lung hilum had also significantly reduced in size, and there was no evidence of invasion into the superior vena cava.

**Figure 4:**
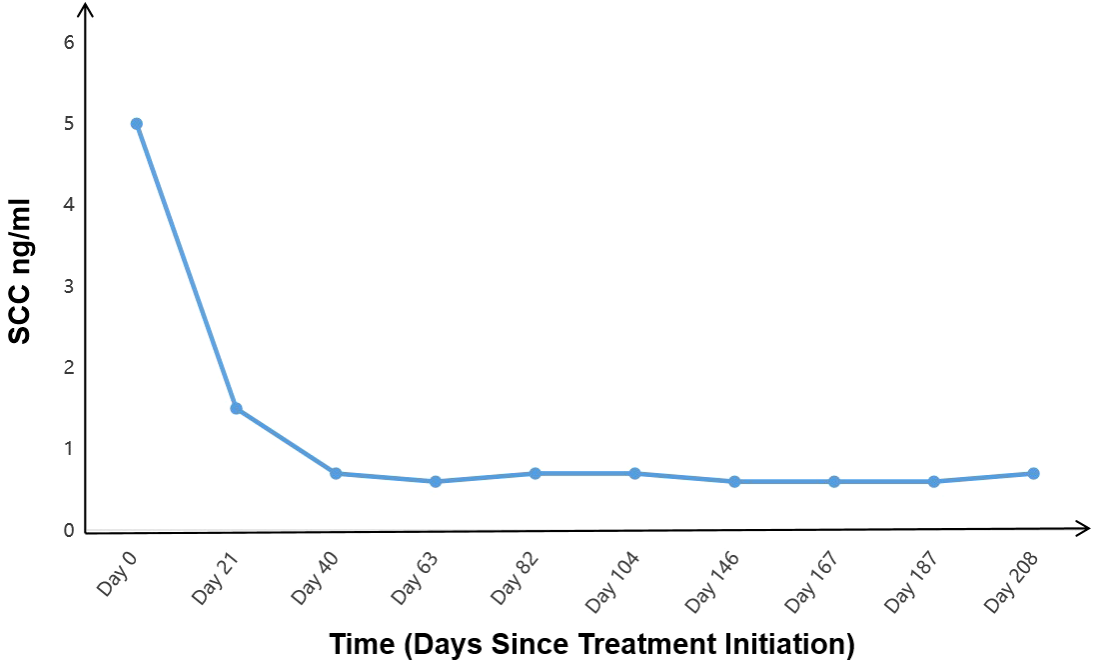
Changes in level of tumor marker SCC during treatment.

**Figure 5:**
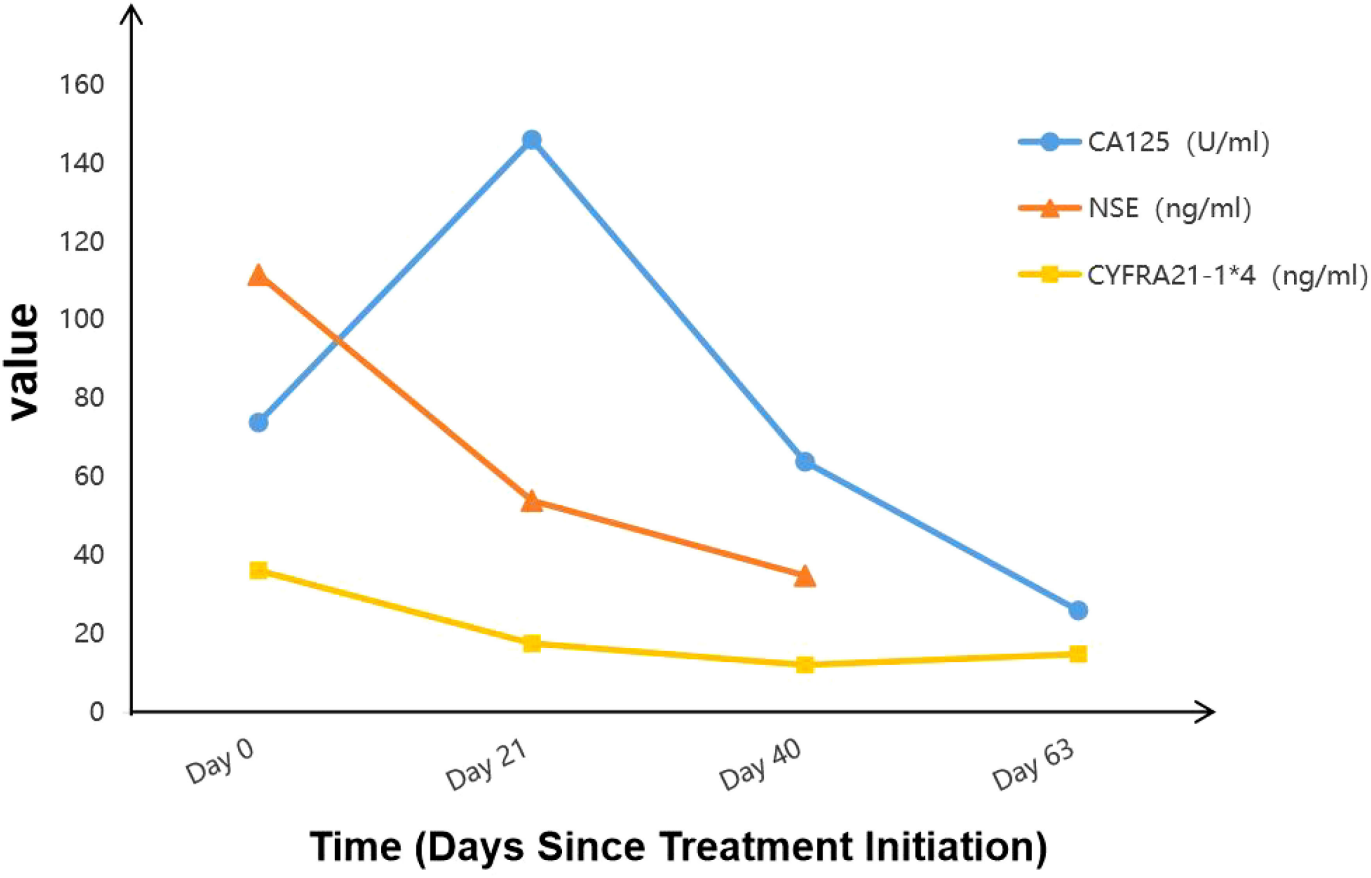
Changes in level of tumor marker CA125, NSE and CYFRA21-1 during treatment.

Additionally, the patient’s squamous cell carcinoma (SCC) antigen levels (**Figure 4**). significantly decreased since the initiation of immunotherapy, and other tumor markers, including carbohydrate antigen 125 (CA125), neuron-specific enolase (NSE), and cytokeratin 19 fragment (CYFRA 21-1), returned to normal levels (**Figure 5**). Notably, CA125 showed a transient increase during the early stages of immunotherapy. Relevant studies suggest that elevated CA125 is not only associated with malignant tumors but can also serve as an auxiliary diagnostic marker for certain inflammatory diseases, potentially related to the systemic immune response triggered by immunotherapy (10).

Throughout the course of immunotherapy, the patient did not experience any significant immune-related adverse events. A follow-up CT scan and other examinations in October 2024 showed further improvement in the consolidation of the right upper lung lobe. The previously enlarged metastatic lymph nodes in the mediastinum, right supraclavicular region, and right hilum remained stable compared to the July 2024 scan, with no new lesions observed. As of January 20, 2025, the patient continues to receive outpatient immunotherapy.

## 3 Discussion

Immunotherapy, by activating the patient’s immune system and enhancing the anti-tumor immune response, has demonstrated significant therapeutic potential (8). Among the various strategies, blocking the PD-1/PD-L1 pathway is currently the primary therapeutic approach. By disrupting the interaction between PD-1 and PD-L1, it inhibits their suppressive effect on T cells and restores the anti-tumor activity of effector T cells, thereby effectively eliminating tumor cells. This mechanism provides a new avenue for the treatment of various cancers (11–13).

For patients with recurrent or metastatic cervical cancer, particularly those whose disease progresses after first-line chemotherapy and have limited treatment options, immunotherapy holds particular promise. Currently, PD-1/PD-L1 immunotherapy is primarily used as a second-line or subsequent treatment option after the failure of first-line therapy (14). Immunotherapy has shown enhanced efficacy, particularly in patients with high PD-L1 expression (15, 16). In this case report, we observed a favorable response in a patient with recurrent and metastatic cervical cancer who had high PD-L1 expression and received Socazolimab immunotherapy. The patient’s metastatic lesions remained stable and continued to shrink, with no new lesions observed. Immune-related adverse events were manageable. These results suggest the potential of Socazolimab in the treatment of advanced recurrent metastatic cervical cancer.

Socazolimab demonstrates a therapeutic advantage over other drugs due to its dual anti-tumor mechanism: it exerts its anti-tumor effect both through antibody-dependent cellular cytotoxicity (ADCC) and by inhibiting programmed death ligand 1 (PD-L1) (9). More importantly, existing studies (NCT03676959) indicate that Socazolimab is effective in patients with varying levels of PD-L1 expression, providing broader opportunities for immunotherapy in patients with recurrent, metastatic advanced cervical cancer.

In this case, the patient showed sustained lesion remission after receiving Socazolimab treatment. While the significant therapeutic effect observed in this patient may be related to the high expression of PD-L1, the specific mechanism of action of the drug still requires further exploration (17). Although this case demonstrated favorable efficacy, we were unable to assess the effect of Socazolimab in a patient with PD-L1 negative expression.

Overall, Socazolimab immunotherapy has shown sustained tumor regression and manageable toxicity in advanced cervical cancer, representing a promising advancement. However, further research is needed to explore the potential mechanisms of the drug’s action and to develop standardized second-line and subsequent treatment regimens. This will provide more treatment options for patients with advanced cervical cancer and potentially improve survival outcomes.

## Data Availability

The datasets presented in this study can be found in online repositories. The names of the repository/repositories and accession number(s) can be found in the article/supplementary material.
